# Serum Galectin-3 and IL-6 as Inflammatory Markers in Bipolar Disorder: Insights from Manic and Euthymic Episodes

**DOI:** 10.3390/jcm14030803

**Published:** 2025-01-26

**Authors:** Alev Lazoglu Ozkaya, Nilifer Gürbüzer, Elif Özcan Tozoğlu, Sumeyya Akyildirim, Filiz Mercantepe

**Affiliations:** 1 Department of Biochemistry, Erzurum City Hospital, 25240 Erzurum, Türkiye; alev.lazogluozkaya@saglik.gov.tr; 2 Department of Psychiatry, Erzurum Faculty of Medicine, University of Health Sciences, 25240 Erzurum, Türkiye; nilifer.gurbuzer@sbu.edu.tr (N.G.); elif.ozcantozoglu@sbu.edu.tr (E.Ö.T.); 3 Department of Psychiatry, Elazig Mental Health and Diseases Hospital, 23100 Elazig, Türkiye; sumeyya.akyildirim@saglik.gov.tr; 4 Department of Endocrinology and Metabolism, Faculty of Medicine, Recep Tayyip Erdogan University, 53100 Rize, Türkiye

**Keywords:** bipolar disorder, Galectin-3, cytokines, inflammation

## Abstract

**Objectives:** This study aimed to assess serum Galectin-3 (Gal-3) and IL-6 levels, along with other inflammatory markers, in type-1 bipolar disorder (BD) patients and explore their relationship with clinical features, metabolic parameters, and symptom severity. **Background:** The study included 38 manic, 35 euthymic BD patients, and 40 healthy controls. Sociodemographic data, such as age, gender, alcohol and smoking habits, and body mass index (BMI), were recorded. **Methods:** The Young Mania Rating Scale (YMRS) and Hamilton Depression Rating Scale (HAM-D) were administered to patients. Biochemical measurements included Gal-3, IL-6, CRP, neutrophil, lymphocyte, platelet counts, and inflammatory indices like NLR, PLR, SII, and SIRI. **Results:** Gal-3 levels significantly differed among the groups (F = 52.251, *p* < 0.001), with the highest levels in euthymic patients. IL-6 levels were elevated in both manic and euthymic patients compared to controls (F = 7.379, *p* = 0.001). Manic patients had significantly higher levels of neutrophils, monocytes, CRP, NLR, PLR, SII, and SIRI. A positive correlation was found between Gal-3 levels, the total number of episodes, and YMRS scores in manic patients. In euthymic patients, Gal-3 levels correlated positively with disease duration and episode count. **Conclusions:** Elevated Gal-3 levels, particularly in the euthymic phase, may serve as a biomarker for BD and indicate ongoing inflammation. These findings suggest Gal-3 could help identify BD and differentiate the euthymic phase.

## 1. Introduction

Bipolar disorder (BD) is a chronic psychiatric disorder characterized by extreme mood swings including manic and depressive episodes. Epidemiologic studies show that bipolar disorder is one of the most important causes of social disability [1]. The emergence and clinical effects of the disorder are shaped by genetic, biological, and environmental conditions. Its complex structure characterized by adverse neuropsychological effects, immunological and physiological changes, and functional disorders makes the diagnosis and treatment processes quite complicated [2].

Research to understand the pathophysiology of bipolar disorder has increased the importance of the relationship between this disorder and inflammation. Inflammation is the body’s immune response and can occur in acute or chronic forms. The acute form is usually triggered by physical conditions such as infection or injury. There are increasing claims that chronic inflammation is associated with many physiological and psychological disorders. In addition to bipolar disorder, the relationship between inflammation and some psychological disorders such as depression, anxiety disorders, and schizophrenia is being investigated [3].

Various studies on systemic inflammation and immune system dysfunction are carried out in the research stages of diseases and basically various blood cells and inflammatory parameters are examined [4]. In addition to the numerical values of some blood cells such as neutrophils, lymphocytes, and monocytes, some parameters such as NLR (Neutrophil/Lymphocyte Ratio), PLR (Platelet/Lymphocyte Ratio), SII (Systemic Immune-Inflammatory Index), and SIRI (Systemic Immune-Inflammatory Reaction Index) have been investigated in patients with BD as in many diseases. Higher neutrophil, lymphocyte, and monocyte counts as well as higher NLR, PLR, SII, and SIRI index values in BD compared to controls are accepted as indicators of systemic inflammation and immune dysfunction [5]. C-reactive protein (CRP), an acute phase reactant produced by the liver, plays a role in the inflammatory response and is a biomarker widely used in the diagnosis and follow-up of systemic inflammation. CRP can be used as an important biomarker in the diagnosis, follow-up, and treatment of bipolar disorder as in other inflammation-related diseases [6]. Increased levels of some pro-inflammatory cytokines (interleukin-1 beta (IL-1β), interleukin-6 (IL-6), tumor necrosis factor-alpha (TNF-α)) have been observed in studies conducted on patients with BD. It has been suggested that these cytokines have neuroinflammatory effects on the central nervous system [7]. Activation of microglial cells, which are defined as immune cells in the brain, plays a critical role in inflammation processes and can increase the release of neuroinflammatory cytokines [8]. Neuroinflammatory cytokines can increase the permeability of the blood–brain barrier, facilitating the entry of peripheral inflammatory cells into the brain, thereby exacerbating inflammatory responses in the brain. The ability of some anti-inflammatory drugs to balance mood symptoms supports the role of inflammation in the pathophysiology of the disease [9]. In bipolar disorder, neuroinflammation caused by increased pro-inflammatory cytokines can affect neurotransmitter systems, leading to mood fluctuations [10]. Additionally, levels of neurotrophic factors such as brain-derived neurotrophic factor (BDNF) may decrease, negatively impacting synaptic functions, neuronal viability, and growth [11]. An increase in pro-inflammatory cytokines can also lead to impairments in cognitive functions [12]. One of the key molecules involved in the regulation of inflammatory responses is Galectin-3 (Gal-3).

The relationship of Gal-3, known to trigger pro-inflammatory molecules and increase inflammation leading to neuroinflammation, with inflammation continues to be investigated [13]. Gal-3 is a β-galactoside-binding lectin belonging to the protein family called galectins. This molecule, which plays an important role in various biological processes, interacts with many cell types in the stages of activating or inhibiting inflammatory mechanisms. In addition to enhancing the activation and chemotaxis of macrophages, Gal-3 promotes the production of pro-inflammatory cytokines such as IL-1β, IL-6, and TNF-α, which play a critical role in initiating and maintaining the inflammatory response [14].

The effects of Gal-3 on chronic inflammation in various psychiatric disorders such as depression [15], schizophrenia [16], anxiety disorders [17], and hyperactivity disorder in children [18] are being investigated. Gal-3 is expressed in various glial cells of the central nervous system tissues, including the brain, both in vivo and in vitro [19]. The support of Gal-3 in the production of anti-inflammatory cytokines in certain situations can be explained by the complex nature of the immune system. In a study conducted in the presence of hypoxic brain injury, Gal-3 was associated with a reduction in the infarct area by increasing the release of anti-inflammatory cytokines [20]. Additionally, another study found that it is synthesized in active microglia, which are effective in axon regeneration and remyelination, and thus plays a role in the clearance of cellular debris and damaged axons, showing effective neuroprotection [21]. It is also suggested that Gal-3 acts as an activator in the early stages of inflammation but inhibits it in later stages [22]. These complex and dynamic interactions indicate that Gal-3 can assume both pro-inflammatory and anti-inflammatory roles. In this context, the role of Gal-3 in bipolar disorder and other psychiatric diseases should be investigated more deeply in terms of modulating inflammatory responses.

The pathophysiological foundations of BD are not fully understood. However, for accurate diagnosis and treatment, it is necessary to thoroughly analyze and understand the underlying mechanisms of the disease. Evaluating the relationship between the severity of the disease and the levels of serum Gal-3, IL-6, and some inflammatory parameters among manic, euthymic, and control groups can contribute to a more detailed analysis of the known neuroinflammation mechanism. Our study aims to examine the differences in clinical characteristics, serum Gal-3, IL-6, and some inflammatory parameters among manic, euthymic, and control groups. Additionally, we evaluated the relationship between Gal-3 and IL-6 levels and the clinical characteristics, duration of the disease, and the Young Mania Rating Scale (YMRS). Specifically, monitoring the levels of new molecules with neuroinflammatory effects and reassessing therapeutic targets through these molecules may open new horizons in the treatment of bipolar disorder.

## 2. Materials and Methods

### 2.1. Participants

This study was designed as an observational case–control study. Patients diagnosed with BD, consisting of a group of 73 adults, who visited the Psychiatry outpatient clinic at Erzurum City Hospital and/or were treated as inpatients in our clinic between May 2024 and July 2024, were included. The control group consisted of individuals without psychiatric disorders who visited our outpatient clinic for consultation or status evaluations between May 2024 and July 2024. All participants and/or their guardians provided written informed consent.

The research protocol was approved by the Scientific Research Ethics Committee of Erzurum Medical Faculty, University of Health Sciences (Erzurum, Turkey) under decision number BAEK 2024/04-85, and was conducted in compliance with the Declaration of Helsinki.

Based on similar studies in the literature and using the G* power statistical program, it was calculated that 102 participants, with 34 in each group, would be required to achieve 80% power and 95% confidence level (effect size = 0.70) [23].

### 2.2. Methods

Our study focused on the cross-sectional clinical characteristics, routine biochemical parameters, and serum Gal-3 and IL-6 analyses of 113 participants. The study included three groups: manic BD patients, euthymic BD patients, and healthy controls. All patients were diagnosed with BD type 1 based on the Diagnostic and Statistical Manual of Mental Disorders, Fifth Edition (DSM-5) [24]. The manic BD group included patients who met the DSM-5 criteria for a manic episode. The euthymic BD group included patients who had not experienced a manic, depressive, or hypomanic episode in the last six months. The control group consisted of individuals without psychiatric disorders who visited the psychiatry outpatient clinic for consultation or status evaluations.

The diagnosis of bipolar affective disorder and the exclusion of additional psychopathologies were made using clinical interviews and the Diagnostic and Statistical Manual of Mental Disorders-5 (DSM-5)-clinician version (SCID-5/CV) [25]. The validity and reliability study of SCID-5/CV was conducted in Turkey [26]. Participants were evaluated twice by two different psychiatrists. Patients completed the YMRS and Hamilton Depression Rating Scale (HAM-D). Clinical and sociodemographic data of the patients were recorded. The control group was formed by individuals without psychopathology, evaluated by psychiatric examination and SCID-5/CV by two different psychiatrists.

In this study, age (years), BMI (kg/m^2^), duration of illness (years), number of episodes (count), and YMRS (score) were identified as independent variables, while Galectin-3 was determined as the dependent variable. These variables were included in the regression model to evaluate the relationships between clinical and demographic factors and Galectin-3 levels.

Inclusion criteria for patients were defined as having no additional psychopathology other than BD-I, being between 18 and 65 years of age, having no acute or chronic physical illness, and not being pregnant or in the postpartum period. Additionally, not receiving pharmacological treatment was required for the manic group, while not using antidepressants or antipsychotics was required for the euthymic group. However, due to ethical concerns and the potential risk of mood episodes if medication was discontinued, the use of classic mood stabilizers such as lithium or valproic acid was not considered an exclusion criterion for the euthymic group.

The inclusion criteria for the control group were being between 18 and 65 years old, having no psychopathology, no acute or chronic medical illness, and not being pregnant or in the postpartum period.

Age (years), gender, BMI, education level, smoking, and alcohol use, which were considered confounding factors, were recorded for all participants using the sociodemographic data form. Body weight was measured using a digital scale, and height was measured with a stadiometer. BMI was calculated by dividing weight in kilograms by the square of height in meters (BMI = kg/m^2^). Patients completed the YMRS and HAM-D. The YMRS was developed by Young and colleagues [27]. It is used not for diagnosis but to determine the severity of the manic state. It consists of 11 items including elevated mood (elevated, inappropriate joking), thought disorder (distractibility; mild distractibility; increased thought production), and each question includes five severity levels. The scale’s validity and reliability have been confirmed for our country, with a Cronbach’s alpha coefficient of 0.79 [28]. The HAM-D scale was completed to assess the severity of depression in patients. It is a 17-item clinician-rated scale developed by Hamilton in 1960 [29]. Items related to difficulty falling asleep, waking during the night, early morning awakening, somatic symptoms, genital symptoms, weight loss, and insight are scored on a scale of 0–2, while other items are scored on a scale of 0–4. It is scored between 0 and 53. A score of 7 or below indicates no depression [30]. The validity and reliability of the Turkish version have been established, with a Cronbach’s alpha coefficient of 0.75 [31].

Participants were asked to rest in a seated position between 08:00 and 10:00. Venous blood samples were taken from the antecubital area for the analysis of Gal-3, IL-6, CRP, and hemogram parameters. Routine blood samples were collected for routine biochemistry parameters, Gal-3, and IL-6 levels. After allowing the samples to clot at room temperature for 30 min, they were centrifuged at 3000 rpm for 10 min to separate the serum. The serum samples were then aliquoted and stored at −80 °C until analysis.

CRP and IL-6 levels were determined using the Siemens Atellica^®^ (Siemens Healthineers, Erlangen, Germany) clinical chemistry analyzer with a spectrophotometric method. Hemogram parameters were measured using the Sysmex^®^ (XN-Series, Kobe, Japan) device in whole blood samples collected in EDTA tubes. Gal-3 measurements in serum samples were analyzed using ELISA kits (BT Lab, Human Galectin-3: Cat Log No: E-EL-H5490, Jiaxing Korain Biotech, Jiaxing, China) according to the manufacturer’s standard protocol on a Rel Assay brand automatic ELISA reader (Biobase Biodusty Co., Ltd., Jinan, China). The measurement range of the kit was 5–2000 pg/mL. After confirming that the serum samples were thawed under appropriate conditions, all analyses were conducted in a single session at the Erzurum City Hospital Medical Biochemistry Laboratory.

### 2.3. Statistical Analysis

The study’s analyses were performed using IBM Statistical Package for the Social Sciences (SPSS) version 22. The normal distribution of continuous variables was evaluated using the Shapiro–Wilk test, Kolmogorov–Smirnov test, Q-Q plot, skewness, and kurtosis data. Data were presented as mean, standard deviation, minimum, maximum, percentage, and frequency. Comparisons between categorical variables were made using the Chi-square test. For comparisons between two independent groups, the Independent Samples *t*-test was utilized since the normality assumption was met. For comparisons of continuous variables among more than two independent groups, the ANOVA test was applied as the normality assumption was satisfied. Post hoc tests following the ANOVA were performed using the Bonferroni test for homogeneous variances and the Tamhane’s T2 test for non-homogeneous variances. Correlation analysis was performed to evaluate the relationship between quantitative variables. The Pearson correlation test was used for comparing two quantitative variables as the normal distribution condition was met. ANCOVA was used for multiple comparisons to examine the effects of co-factors on the dependent variable. Receiver operating characteristic (ROC) curve analysis was conducted to assess the diagnostic potential of continuous variables and to establish cut-off values. A *p*-value of less than 0.05 was considered statistically significant.

Regression analysis is a statistical method used to model and evaluate the relationship between a dependent variable and one or more independent variables. It helps to understand how the dependent variable is influenced by the independent variables, measure this effect, and predict future values. In our study, linear regression analysis was used to assess the impact of independent variables on the dependent variable (Galectin-3).

## 3. Results

The study included 73 patients with type 1 bipolar disorder of similar age and gender, and 40 healthy controls. Of the 73 patients included in the study, 38 were in a manic episode and 35 were in a remission period. The sociodemographic and clinical characteristics of the patient and control groups are presented in Table 1.

In the comparison of the manic period, euthymic period, and control group, neutrophil, monocyte, CRP, NLR, PLR, SII, and SIRI levels were found to be significantly higher in manic period patients compared to the other groups. There was a significant difference in Gal-3 levels among the three groups. The highest Gal-3 levels were in patients in the euthymic period. IL-6 levels were higher in both manic and euthymic period patients compared to the controls (Table 2).

**Figure 1:**
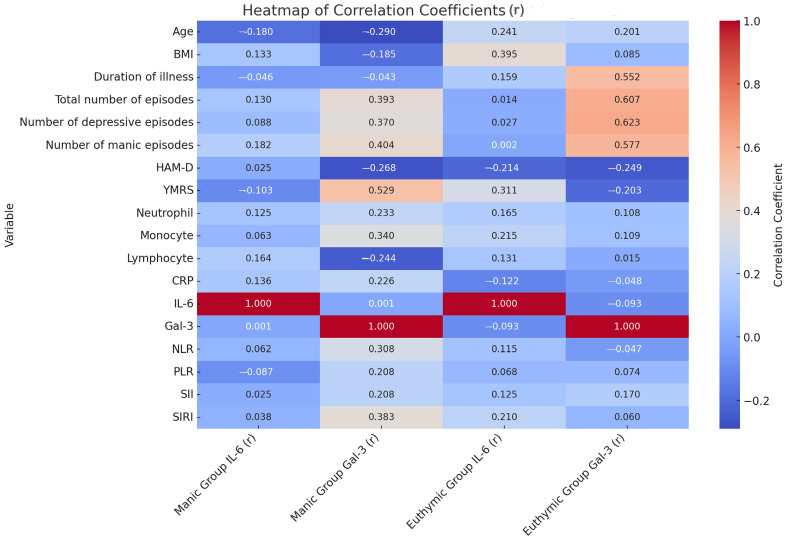
Relationship between clinical data of patients and hemogram and biochemical parameters. r: correlation coefficient; BMI: Body Mass Index; CRP: C-Reactive Protein; Gal-3: Galectin-3; HAM-D: Hamilton Depression Rating Scale; IL-6: Interleukin-6; NLR: Neutrophil/Lymphocyte Ratio; PLR: Platelet/Lymphocyte Ratio; SII: Systemic Immune Inflammation Index; SIRI: Systemic Inflammatory Response Index; YMRS: Young Mania Rating Scale.

When the correlations between Galectin-3 and inflammatory markers (IL-6, neutrophil, monocyte, lymphocyte, CRP, NLR, PLR, SII, and SIRI) were examined, generally weak and statistically non-significant relationships were observed in both groups. In the manic group, a low-level positive significant correlation was found between Galectin-3 and monocyte levels (r = 0.340, *p* = 0.037). Apart from this, no significant correlations were observed with other inflammatory markers. In the euthymic group, no significant correlation was detected between Galectin-3 and any inflammatory marker. The Gal-3 levels in patients during the manic period showed a moderate significant positive correlation with the total number of episodes (r = 0.393, *p* = 0.15) and the total YMRS score (r = 0.529, *p* = 0.01). The Gal-3 level was significantly and positively correlated with both the number of manic and depressive episodes. In patients during the euthymic period, the Gal-3 level showed a moderate significant positive correlation with the duration of the disease (r = 0.552, *p* = 0.01) and the total number of episodes (r = 0.607, *p* = 0.000) (Figure 1). This suggests that Galectin-3 may be more strongly associated with clinical parameters such as the frequency of episodes and the duration of the illness rather than with inflammatory markers.

**Figure 2:**
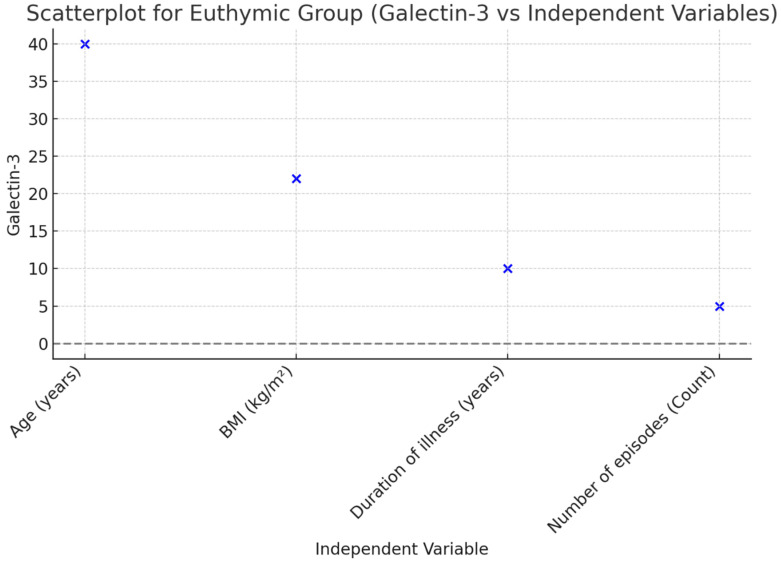
Scatterplot illustrating the relationship between Galectin-3 and independent variables in the Euthymic Group.

**Figure 3:**
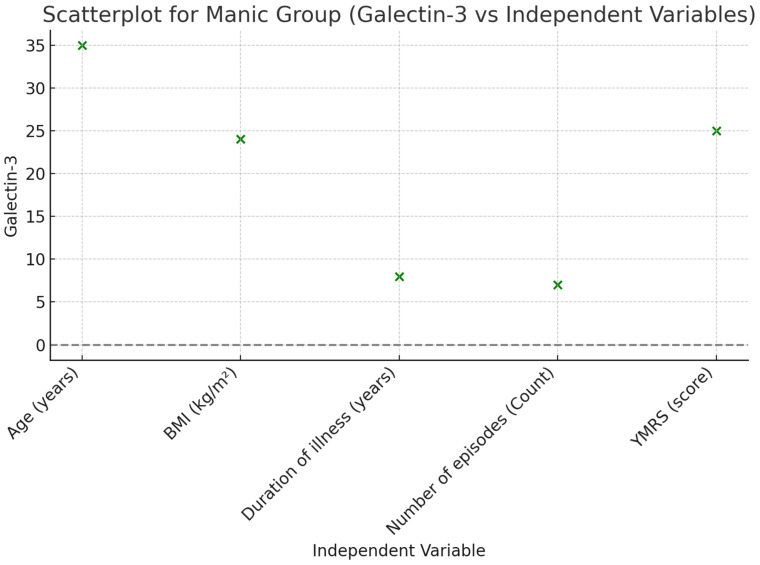
Scatterplot illustrating the relationship between Galectin-3 and independent variables in the Manic Group.

A linear regression analysis was performed to examine the effects of common variables in a mixed model, including age, BMI, disease duration (years), and the number of episodes in patients during the euthymic period. According to the analysis results, it was found that disease duration (B ± SE; 23.157 ± 10.063, *p* = 0.029) and the number of episodes (B ± SE; 53.931 ± 18.965, *p* = 0.008) were effective in the model created for Gal-3. A linear regression analysis was conducted to assess the effects of common variables in a mixed model, including age, BMI, disease duration (years), number of episodes, and YMRS score in patients during the manic episode period. According to the analysis results, it was found that the number of episodes (B ± SE; 55.016 ± 21.100, *p* = 0.014) and YMRS score (B ± SE; 26.873 ± 8.121, *p* = 0.002) were effective in the model created for Gal-3 (Table 3, Figure 2 and Figure 3).

**Figure 4:**
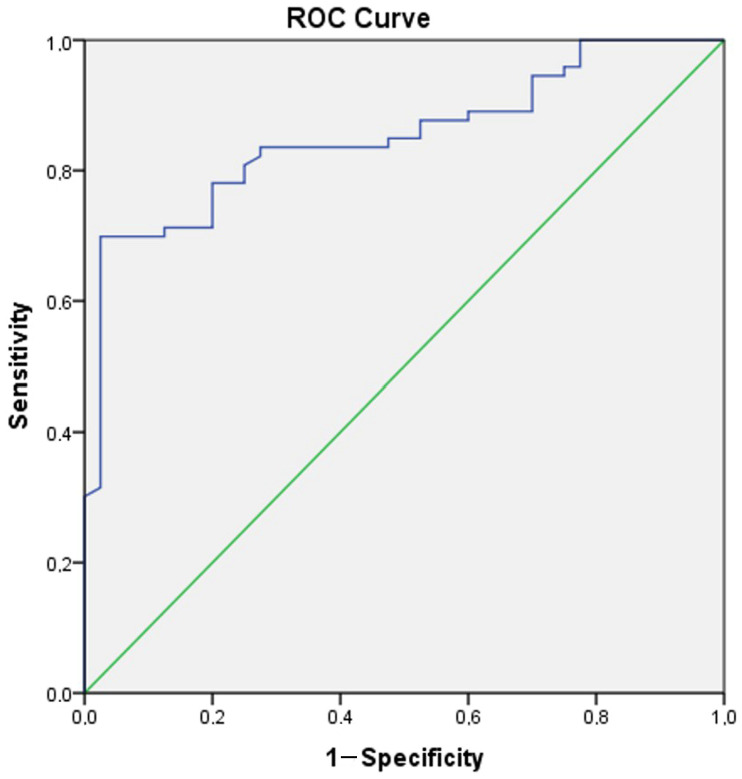
ROC curve analysis for Galectin-3 (Patient group–Control group). Galectin-3 cut off value (Patient group–Control group): 829.58, sensitivity 78.1%, specificity 80%.

**Figure 5:**
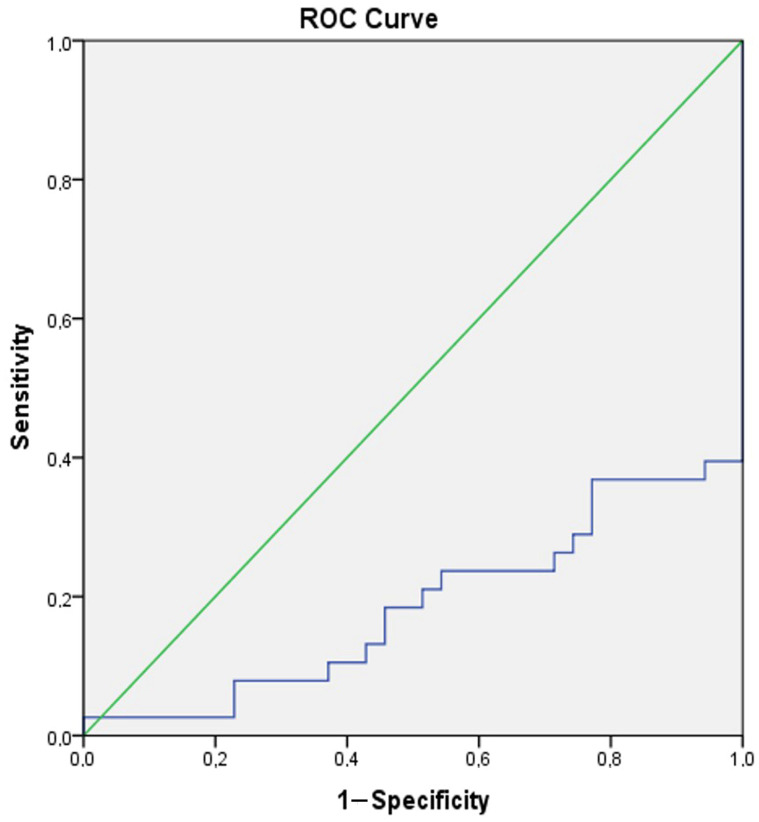
ROC curve analysis for Galectin-3 (Manic group–Euthymic group). Galectin-3 cut off value (Manic group-Euthymic group): 1189.64, sensitivity 71.1%, specificity 77.1%.

A ROC analysis was conducted to reveal the role of Gal-3 in distinguishing manic and euthymic periods in both the control group and patients, and to determine the cut-off values. The analysis results showed that Gal-3 (AUC ± SE; 95% CI; 0.695 ± 0.054; 0.590–0.801) can be used to identify BD and that higher values increase the likelihood of identifying the presence of BD (Table 4, Figure 4). Additionally, it was shown that Gal-3 (AUC ± SE; 95% CI; 0.695 ± 0.054; 0.590–0.801) can also be used as a period marker in patients (Table 4, Figure 5).

## 4. Discussions

The presence of increased pro-inflammatory cytokines and neuroinflammation in patients with BD has been acknowledged [7,10]. We hypothesized that there would be disruptions in the regulation of Gal-3, a molecule closely related to inflammation, and IL-6, a pro-inflammatory cytokine, in BD patients both compared to controls and between manic and euthymic periods. In this study, we assessed the differences in serum Gal-3 and IL-6 levels among manic BD patients, euthymic BD patients, and healthy controls, as well as their relationship with clinical characteristics, various hematological parameters, symptom severity, and psychopathology. Our results indicated that Gal-3 and IL-6 levels were elevated in both manic and euthymic BD patient groups compared to healthy controls. We observed significant differences in serum Gal-3 levels among BD patients, with the highest levels found in the euthymic patient group. We revealed that the number of episodes and the severity of the episode were effective on Gal-3 in patients during the manic period. Additionally, we found that the number of episodes and the duration of the disease were effective on Gal-3 in patients during the euthymic period. Our results indicated that Gal-3 is a marker for both the disease and the period.

In our study, the use of antipsychotics was an exclusion criterion during the manic phase. Patients in the manic phase and controls were not using medication. However, the use of mood stabilizers by patients in the euthymic phase can be considered a confounding factor. The effect of lithium, one of the most commonly used mood stabilizers, on inflammation is controversial [32].

In our study, Gal-3 levels showed significant differences among the three groups. We found the highest Gal-3 levels in patients in the euthymic phase and the lowest levels in healthy controls. It is known that Gal-3 can contribute to neuroinflammation through pro-inflammatory cytokines [13], affect mood fluctuations by influencing neurotransmitter systems [10], and cause negative effects on synaptic plasticity and neuronal survival by reducing levels of neurotrophic factors such as brain-derived neurotrophic factor (BDNF) [11]. The presence of neuroinflammation has also been associated with impairments in cognitive functions [12]. The ability of Gal-3 to support the production of anti-inflammatory cytokines in some cases reflects the complex nature of the immune system [20]. Some studies suggest that Gal-3 exhibits a pro-inflammatory effect during the early stages of inflammation and shifts to an anti-inflammatory role during the recovery phase [22]. In our study, we observed that Gal-3 levels increased in the manic attack patient group due to its pro-inflammatory effect and continued to rise, peaking during the euthymic phase, even though the patients were in remission. This situation was thought to be due to the anti-inflammatory effect of Gal-3 in the later stages of the disease and consequently inflammation.

It is widely accepted that many psychiatric disorders are chronic and associated with neuroinflammation [15,16,17,18]. Existing literature data reveal that Gal-3 levels are elevated in various neuropsychiatric disorders and function as a modulator of inflammatory responses. For instance, a study on children with autism spectrum disorders found that Gal-3 levels were significantly higher compared to healthy controls. They suggested that Gal-3 plays a central role in inflammation and neuroinflammation processes [33]. A study investigating the predictive power of Gal-3 for post-stroke cognitive impairment found that serum Gal-3 levels were significantly higher in the patient group and that higher levels were proportional to the likelihood of developing post-stroke cognitive impairment [34].

Similarly, there are studies showing that Gal-3 levels are elevated in neurodegenerative diseases. A study emphasized the significance of elevated Gal-3 levels in diseases like Alzheimer’s, Parkinson’s, and amyotrophic lateral sclerosis, which are known to be associated with neuroinflammation [35]. Additionally, in an animal experiment, the suppression of Gal-3 in Alzheimer’s disease resulted in improved cognitive performance, reduced expression of inflammatory genes in microglia, and regression of amyloid plaques [36]. A study conducted on patients with frontotemporal dementia found that increased Gal-3 levels were associated with cognitive decline in the disease. This finding suggests that Gal-3 may play a role in neuroinflammatory processes in patients with frontotemporal dementia and could be considered a biomarker and therapeutic target for this disease [37]. Another animal study investigated the role of Gal-3 in modulating anxiety levels. In mice with the Gal-3 gene removed, IL-6 increase was prevented, and hippocampal GABA levels decreased. These findings suggest that Gal-3 may modulate anxiety levels by enhancing inflammatory responses [17].

According to the results of our study, one of the most interesting findings was that Gal-3 levels were higher in patients during the euthymic phase compared to those in the manic phase. The elevated Gal-3 levels observed in patients during the euthymic phase, as noted in the literature, may be attributed to the anti-inflammatory effect of Gal-3 in the later stages of inflammation [22]. This situation can be suggested to be for the purpose of repairing tissues, nerve cells, and synaptic connections affected by inflammation, reorganizing the immune system, and maintaining homeostasis. Additionally, the fact that Gal-3 levels are significantly higher not only between the euthymic and manic phases but also compared to the control group supports the idea that it could be a biomarker specific to both the disease and the phase. Another study conducted with BD patients found that Gal-3 levels were statistically significantly higher at the end of the third week of treatment compared to the first day of hospitalization, supporting our findings that Gal-3 is related to the disease and its inflammatory processes. Furthermore, the higher Gal-3 levels obtained in the third week of treatment suggest that the anti-inflammatory mechanisms of this molecule play a role in the post-attack processes, as in our study [23].

In our study, it was notable that IL-6 levels were significantly higher in bipolar disorder patients compared to healthy controls, even though there was no significant difference between the manic and euthymic phases. Studies conducted on BD patients have observed increases in the levels of certain pro-inflammatory cytokines, along with neuroinflammatory effects. The finding that IL-6 levels are significantly elevated in both the manic and euthymic phases of bipolar disorder compared to healthy controls suggests that the disease has an inflammatory component, with inflammation playing a role across all phases of the disorder. While increased stress and biological activity during manic phases suggest the presence of acute inflammatory responses, it can be accepted that inflammatory responses continue in the euthymic phase due to the chronicity of inflammation. These findings suggest that IL-6 could be used as a biomarker in the treatment and monitoring of bipolar disorder. IL-6 levels can be monitored to evaluate treatment response and the risk of disease exacerbation. Anti-inflammatory treatment strategies may be helpful in the management of bipolar disorder, and reducing IL-6 levels could be targeted.

In our study, it was important to note that neutrophil and monocyte counts, inflammation indices such as NLR, PLR, SII, and SIRI, and CRP values were significantly higher in the manic phase compared to euthymic and control patients. Studies have also interpreted the relationship between these parameters and BD disease based on the presence of systemic and neuroinflammation. Our study shows that the pro-inflammatory effects of Gal-3 in the manic phase become prominent in a manner consistent with the other inflammatory parameters we investigated. This consistency also proves how effective inflammation is in the manic phase of BD disease.

Our results show that Gal-3 levels in bipolar disorder may be associated with inflammation and neuroinflammation. High Gal-3 levels in both manic and euthymic phases compared to controls indicate that the disease has an inflammatory component, and that this protein plays a role in both phases of the disease. However, the higher Gal-3 levels we detected in the euthymic phase bring a unique dimension to this study. Elevated Gal-3 levels, particularly in euthymic patients, may be useful in assessing the disease course and treatment response, given their close association with the number of episodes and disease duration. The significant increase in levels, closely related to the duration of the disease in the euthymic period, indicates that the expected anti-inflammatory effect of Gal-3 will come to the forefront in the later stages. These findings not only support the potential use of Gal-3 as a biomarker for BD disease but also strongly suggest its potential as a phase marker for the euthymic period, which is the most difficult and complex period to diagnose, especially at the initial presentation. We can propose that our study is one of the important steps in developing new targets for accurate treatment, including Gal-3. All findings also emphasize the importance of further research in this area.

This study has both strengths and limitations. Due to its cross-sectional design, it is difficult to determine the direction of causality between variables. Another limitation is the lack of simultaneous radiological evaluations and the inability to examine all inflammation-related molecules. Additionally, the absence of further analysis on the type and dosage of mood stabilizers used by the euthymic group and their relationship with inflammatory markers is considered a limiting factor. Studies with blood samples collected from the same patients during both attack and remission periods and with larger sample sizes could provide more insights into the role of Gal-3 in bipolar disorder.

On the other hand, our study presented valuable results showing the difference in increased serum Gal-3 levels between phases in patients with BD. Additionally, the use of diagnostic evaluations based on structured clinical interviews, the Turkish validity and reliability of the scales employed, the assessment of participants by two independent psychiatrists, the evaluation of multiple molecules alongside Gal-3 and IL-6, and the selection of a well-matched control group are other strengths of this research.

## 5. Conclusions

In this study, we evaluated the possible role of Gal-3 in the pathophysiology of the disease by examining Gal-3 levels in manic and euthymic phases in bipolar disorder. Our findings indicate that Gal-3 levels are significantly elevated in both the manic and euthymic phases compared to healthy controls, with levels being notably higher in the euthymic phase than in the manic phase. The elevated Gal-3 levels in the euthymic phase were found to be associated with the duration of the disease and the number of episodes. With these results, we believe that high Gal-3 levels will shed light on the complex inflammation process in BD, especially in the euthymic phase, from a different perspective. It can be suggested that Gal-3 could be an indicator and/or structural cause of the disease and a state marker for the euthymic phases.

We believe that investigating Gal-3 and IL-6 levels in BD will be promising for future studies to better understand neuroinflammation.

## Figures and Tables

**Table 1:** Comparison of sociodemographic and clinical characteristics of the manic period, euthymic period, and control groups.

		Manic Group n%	Euthymic Group n%	Control Group n%	Chi-Square/F	*p*
Age (year)	Mean ± SD	37.55 ± 13.12	40.31 ± 9.58	38.4 ± 11.53	0.543	0.583
Gender	Female	18 (47.4)	17 (48.6)	15 (37.5)	1.154	0.562
	Male	20 (52.6	18 (51.4)	25 (62.5)		
Marital status	Single	17 (44.7)	14 (40)	13 (32.5)	6.922	0.135
	Married	18 (47.4)	15 (42.9)	26 (65)		
	Widowed, Divorced, Living apart	3 (7.9)	6 (17.1)	1 (2.5)		
Occupation	Public employee	5 (13.2)	7 (20)	37 (92.5)	63.111	<0.001
	Private sector employee	6 (15.8)	8 (22.9)	2 (5)		
	Unemployed	27 (71)	20 (57.2)	1 (2.5)		
Educational status	Primary school	14 (36.)	17 (48.6)	2 (5)	51.535	<0.001
	High school	21 (55.3)	13 (37.1)	8 (20)		
	University	3 (7.9)	5 (14.3)	30 (75)		
Smoking	Yes	22 (57.9)	29 (82.9)	17 (42.5)	12.811	0.002
	No	16 (42.1)	6 (17.1)	23 (57.5)		
Alcohol use	Yes	2 (5.3)	0 (0)	5 (12.5)	4.710	0.081
	No	36 (94.7)	35 (100)	35 (87.5)		
Presence of mental illness in the family	Yes	2 (5.3)	5 (14.3)	4 (10)	1.689	0.486
	No	36 (94.7)	30 (85.7)	36 (90)		
Drug use	Lithium		11 (31.4)			
	Valproic Acid		14 (40)			
	Other Mood Stabilizers		10 (28.6)			

*p* < 0.05 is statistically significant; F: one-way ANOVA test value; Mean ± SD: Mean ± standard deviation; n: number of participants.

**Table 2:** Comparison of clinical characteristics, Gal-3 and IL-6 levels, and hemogram and biochemical parameters of the manic period, euthymic period, and control groups.

	Manic Group n = 38	Euthymic Group n = 35	Control Group n = 40	t/F	Effect Size/Eta Squared	*p*
	**Mean ± SD**	**Mean ± SD**	**Mean ± SD**			
Duration of illness (year)	14.55 ± 10.71	14.20 ± 6.48		−0.172	0.039	0.864
Total number of episodes	5.87 ± 3.78	6.66 ± 3.13		0.966	0.227	0.337
Number of depressive episodes	2.82 ± 2.25	3.31 ± 1.56		1.089	0.253	0.280
Number of manic episodes	3.05 ± 1.61	3.34 ± 1.61		0.770	0.180	0.444
HAM-D	0.79 ± 6.39	1.05 ± 0.87		1.284	0.057	0.203
YMRS	38.63 ± 6.39	1.29 ± 1.10		−35.461	8.144	<0.001
BMI (kg/m^2^)	27.83 ± 4.08 ^a^	30.83 ± 3.97 ^b^	26.19 ± 3.90 ^ac^	12.868	0.190	<0.001
Neutrophil (10^9^/L)	6.12 ± 1.84 ^a^	4.57 ± 1.65 ^b^	4.03 ± 1.44 ^bc^	16.699	0.233	<0.001
Monocyte (10^9^/L)	0.72 ± 0.24 ^a^	0.58 ± 0.19 ^b^	0.60 ± 0.14 ^bc^	5.718	0.094	0.004
Lymphocyte (10^9^/L)	1.95 ± 0.69 ^a^	2.88 ± 0.92 ^b^	2.35 ± 0.63 ^ac^	14.294	0.206	<0.001
CRP (mg/L)	6.16 ± 4.73 ^a^	3.50 ± 3.49 ^b^	2.87 ± 2.11 ^bc^	9.091	0.142	<0.001
IL-6 (pg/mL)	3.66 ± 2.22 ^a^	3.91 ± 2.51 ^ab^	2.29 ± 1.03 ^c^	7.379	0.118	0.001
Gal-3 (pg/mL)	989.47 ± 418.02 ^a^	1469.66 ± 340.06 ^b^	686.01 ± 213.17 ^c^	52.251	0.487	<0.001
NLR	3.58 ± 1.71 ^a^	1.82 ± 1.06 ^b^	1.82 ± 0.81 ^bc^	25.201	0.314	<0.001
PLR	148.70 ± 47.18 ^a^	103.83 ± 49.28 ^b^	123.29 ± 33.77 ^bc^	9.736	0.150	<0.001
SII	942.53 ± 491.08 ^a^	503.18 ± 362.71 ^b^	495.40 ± 235.62 ^bc^	17.537	0.242	<0.001
SIRI	2.69 ± 1.62 ^a^	1.06 ± 0.69 ^b^	1.08 ± 0.52 ^bc^	29.132	0.346	<0.001

*p* < 0.05 is statistically significant in group comparison; effect size = Cohen’s d (0.2-small, 0.5-medium, and 0.8-large effect size); eta squared: 0.01-small, 0.06-medium, and 0.14-large effect size; F: one-way ANOVA value; ^a–c^: significance among the three groups; HAM-D: Hamilton Depression Rating Scale; CRP: C-reactive protein; IL-6: Interleukin-6; NLR: Neutrophil/Lymphocyte ratio; PLR: Platelet/Lymphocyte ratio; SII: Systemic Immune Inflammation Index; SIRI: systemic inflammatory response index; n: number of participants; BMI: Body Mass Index; YMRS: Young Mania Rating Scale. Mean ± SD: mean ± standard deviation.

**Table 3:** Linear regression analysis for Gal-3 in patients with bipolar affective disorder in remission and manic episodes.

Parameters	Independent Variables	B (95% CI)	*p* Value	R^2^/Adjusted R^2^	*p* Value for F Change
Galectin-3 (Euthymic Group)	Model			0.478/0.408	0.000
	Age (years)	−10.933 (−23.302/1.437)	0.081		
	BMI (kg/m^2^)	−5.967 (−29.895/17.961)	0.614		
	Duration of illness (years)	23.157 (2.606/43.709)	0.029		
	Number of episodes (Count)	53.931 (15.200/92.662)	0.008		
Galectin-3 (Manic Group)	Model			0.534/0.461	0.000
	Age (years)	−12.845 (−30.184/4.494)	0.141		
	BMI (kg/m^2^)	−1.999 (−29.144/25.145)	0.882		
	Duration of illness (years)	−0.979 (−26.765/24.807)	0.939		
	Number of episodes (Count)	55.016 (12.036/97.996)	0.014		
	YMRS (score)	26.873 (10.331/43.415)	0.002		

*p* < 0.05 is statistically significant; B: regression coefficients; BMI: Body Mass Index; CI: Confidence Intervals; YMRS: Young Mania Rating Scale.

**Table 4:** Area under the curve.

Test Result Variable(s)		Area	Std. Error ^a^	Asymptotic Sig. ^b^	Asymptotic 95% Confidence Interval	
					Lower Bound	Upper Bound
Patient group (total)-Control group	Galectin-3	0.851	0.036	0.000	0.781	0.921
Manic group-Euthymic group	Galectin-3	0.814	0.051	0.000	0.715	0.914

*p* < 0.05 is statistically significant; ^a^, Under the nonparametric assumption; ^b^, Null hypothesis; true area = 0.5.

## Data Availability

All data generated or analyzed during this study are included in this article. The data will be available upon reasonable request (contact persons: filiz.mercantepe@saglik.gov.tr).
